# The Hook Effect: A Case Study of a Giant Invasive Prolactinoma With Falsely Low Serum Prolactin

**DOI:** 10.7759/cureus.46194

**Published:** 2023-09-29

**Authors:** Rafaela F Gonçalves, Marco Antônio S Vaz, Guilherme Rollin, Gustavo Rassier Isolan

**Affiliations:** 1 Neurology, The Center for Advanced Neurology and Neurosurgery (CEANNE), Porto Alegre, BRA; 2 Neurosurgery, The Center for Advanced Neurology and Neurosurgery (CEANNE), Porto Alegre, BRA; 3 Neurological Surgery, Hospital Moinhos de Vento, Porto Alegre, BRA

**Keywords:** prolactin-secreting adenoma, neuro-surgery, macroprolactinoma, prolactinoma, hook effect

## Abstract

Prolactinomas are benign pituitary tumors also known as prolactin-secreting adenomas (PSA). These tumors cause excessive secretion of prolactin (hyperprolactinemia), a hormone responsible for lactation. Diagnosing hyperprolactinemia relies on measuring prolactin levels in the blood, and elevated serum levels of prolactin are typically indicative of prolactinoma. The hook effect occurs in immunological tests such as the prolactin level test. When the amount of prolactin present in the sample is too high and exceeds the binding capacity of the antibodies being used, the test result may indicate falsely low levels of prolactin, which is the hook effect. The present study describes the case of a male patient who presented with neck pain and difficulty swallowing. MRI revealed a giant (>40mm) extradural tumor affecting the clivus, anterior fossa, pterygopalatine, and bilateral infratemporal fossae as well as the petrous apex and bilateral cavernous sinuses. Endocrinological investigation yielded no specific abnormalities. An occipitocervical fixation (arthrodesis) was proposed with simultaneous extended endoscopic endonasal resection. Surgery succeeded in resecting a portion of the clival tumor and the anterior fossa. Measurement of prolactin levels several weeks post-surgery found them to be extremely high, confirming the hook effect.

## Introduction

Prolactinomas are benign pituitary tumors also known as prolactin-secreting adenomas (PSA). These tumors are more common in women of childbearing age and have an incidence of three to five per 100,000 inhabitants; however, these values may vary depending on the region [1]. It causes excessive secretion of prolactin (hyperprolactinemia), a hormone responsible for lactation. Diagnosing hyperprolactinemia relies on measuring prolactin levels in the blood, and elevated serum levels of prolactin are typically indicative of a prolactinoma [1-3]. However, it is important to consider the hook effect, which can occur in cases of extremely high prolactin concentrations [4].

The hook effect is seen in laboratory assays using antibodies to detect and quantify a substance [4,5] and when antibody saturation renders a falsely low result. In a typical assay, antibodies are used to bind to the substance of interest, forming a measurable complex. However, when there is an excessive concentration of the substance, the available antibodies are overwhelmed and cannot bind to all of the molecules of the substance [4-6]. Some portion of the substance remains unbound to antibodies and does not contribute to the measured signal. This leads to a falsely low measurement of the concentration of the substance, possibly resulting in misdiagnosis [4]. The hook effect occurs in very high levels of prolactin; however, false low levels can also happen in normal levels of the hormone.

To mitigate the hook effect and obtain accurate prolactin measurements, dilution of the blood sample is recommended. Dilution reduces the concentration of prolactin, thus enabling accurate measurement. In suspected cases of the hook effect, healthcare providers may request a dilution study or employ alternate testing methods, such as serial dilutions or other specialized assays, to confirm the diagnosis of hyperprolactinemia [4-6].

## Case presentation

**Figure 1:**
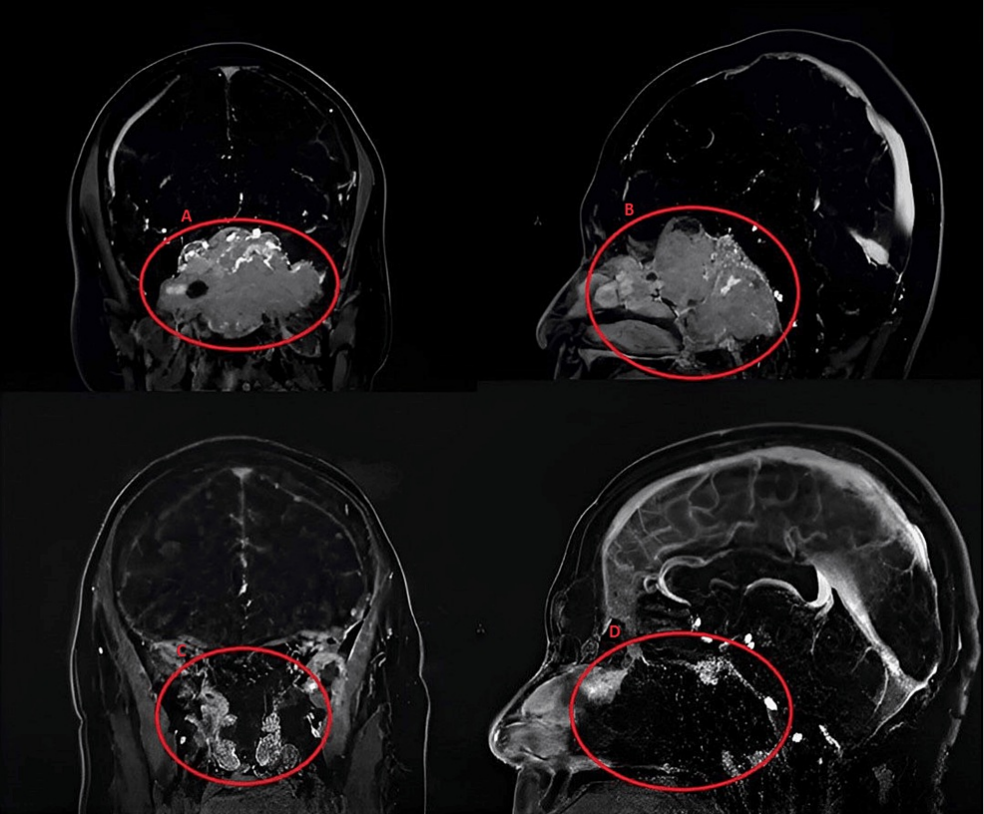
Coronal and sagittal brain MRI axial T1-weight contrast-enhanced showing a large clival tumor (A and B) that was resected in the middle line (C and D).

A male patient presented with a giant extradural tumor affecting the clivus, anterior fossa, pterygopalatine, and bilateral infratemporal fossae, as well as the petrous apex and bilateral cavernous sinuses (Figure 1). The patient presented with recent onset cervical pain, as well as difficulty swallowing and hoarseness due to diagnosed vocal cord paralysis found during laryngoscopy. Furthermore, the patient reported mild atrophy in the lower limbs due to tropical spastic paraparesis (TSP) diagnosed in adolescence. There were no reported visual symptoms, cranial nerve abnormalities, or signs of hyperprolactinemia such as galactorrhea, changes in sexual characteristics, hirsutism, acne, etc. Endocrinological investigation showed a normal prolactin level of 17 Ng/ml, and no other abnormalities were present, The dilution test was not performed initially because, with the large volume that was resected from the tumor, we expected a significant decrease in prolactin. Investigation of the craniovertebral junction (CVJ) revealed instability due to occipital condyle erosion. There was also a history of hereditary spastic paraplegia.

**Figure 2:**
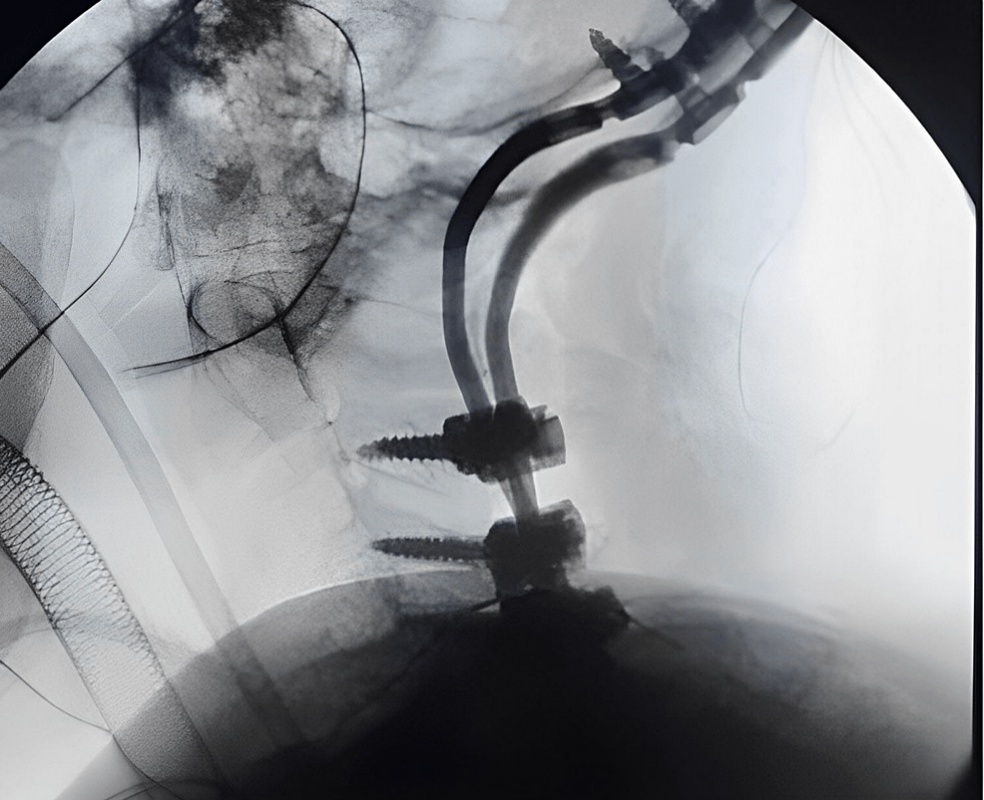
Intraoperative image of occiptocervical fusion.

The patient underwent occipitocervical fixation (arthrodesis) (Figure 2). Simultaneous extended endoscopic endonasal resection was performed with the assistance of neuronavigation and intraoperative neurophysiological monitoring. The surgery aimed to resect the clival portion of the tumor and the skull base involving the anterior fossa (Figure 1).

The surgery was successful, and the patient was discharged without a nasoenteral tube after evaluation by a videofluoroscopic swallowing study and laryngoscopy, which demonstrated effective swallowing without bronchoaspiration. An MRI performed 24 hours after surgery confirmed total resection of the clival component and the anterior fossa involving the internal carotid arteries, as planned prior to surgery (Figure 1).

**Figure 3:**
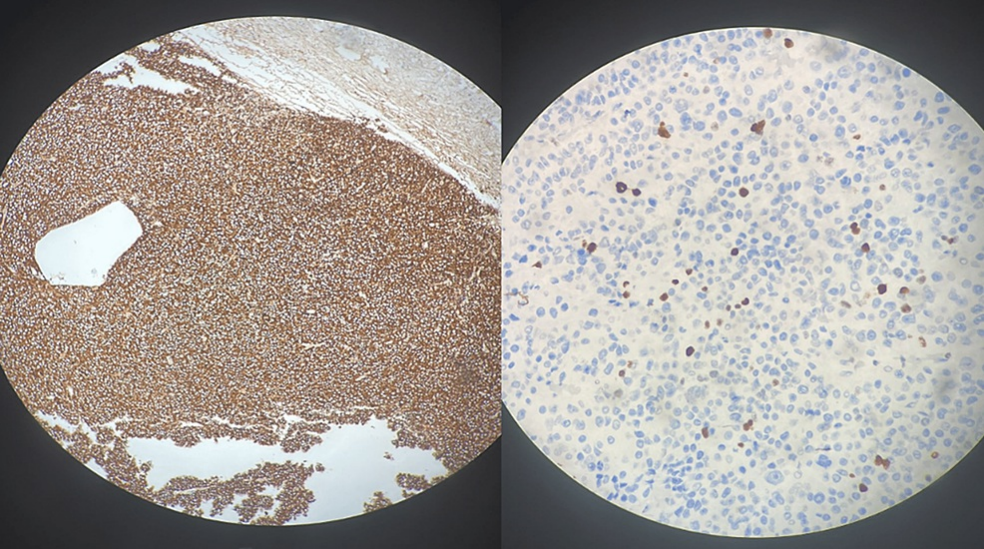
Prolacine was immunopositive in 100 % of the cells (left) while Ki67 was positive in 10% of the cells.

A histopathological examination and an immunohistochemical study, together with the MRI findings, confirmed the diagnosis of invasive adenoma arising from prolactin-producing cells, or macroprolactinoma. Prolactin was positive in 100% of the cells and Ki-67 was positive in 10% of the cells (Figure 3).

Subsequent measurement of prolactin levels weeks after the surgery was found to be at 470 Ng/ml, thus confirming the hook effect.

Based on this diagnosis, cabergoline was started and a positron emission tomography (PET) scan was done. There was no tumor present elsewhere, confirming the diagnosis of invasive adenoma rather than carcinoma. After the pharmacological treatment, prolactin levels dropped to 170 Ng/ml.

## Discussion

The hook effect is observed in immunological tests, such as when measuring serum levels of prolactin, where the results can be falsely low due to excessive antibody saturation [7]. This occurs when the level of prolactin in the sample is so high that it exceeds the binding capacity of the antibodies used in the test. As a result, the test may indicate falsely low levels of prolactin, leading to incorrect diagnoses or underestimation of the severity of certain conditions such as prolactinomas [4,5,7]. Other conditions can also increase prolactin levels and deserve attention, such as hypothyroidism where levels typically exceed 50 ng/mL, chronic kidney failure presenting elevated prolactin, albeit below 100 ng/mL, and the use of medications such as benzodiazepines, phenytoin, metoclopramide, etc., where the hormone levels are in a range above 50 ng/mL [3]. Prolactinomas are benign pituitary tumors that excessively secrete the hormone prolactin [8], thus contributing to the hook effect. These tumors can vary in size and invasiveness and, in rare cases, become giant, invasive prolactinomas.

Initially, prolactinomas are pharmacologically treated using dopaminergic agonists. The first-choice drug is cabergoline with an initial dose of 0.25 mg twice weekly as a starting dose, increasing by 0.25 mg per week, up to 1 mg orally twice weekly in four weeks, maintaining as the serum prolactin level drops [3].

Previous studies have addressed the hook effect in different clinical contexts [9,10]. For example, a case study published by Smith et al. describes a patient with a giant and invasive prolactinoma who initially presented with serum levels of prolactin found to be low [9]. However, after performing a serial dilution test, the hook effect was observed when results indicated significantly higher serum levels of prolactin. This demonstrates the importance of accounting for the hook effect when evaluating prolactin test results in cases of prolactinomas.

Recent studies have investigated alternative approaches to overcome the hook effect and obtain more accurate results [11,12]. For instance, a study conducted by Baranski et al. proposed the use of multi-dilution immunoassays to avoid the hook effect in giant prolactinomas [11]. The results of this study showed a more precise correlation between tumor size and serum prolactin levels, highlighting the importance of these strategies for a more accurate assessment of hormonal status in these patients.

A pituitary adenoma is very hard to diagnose as it can mimic many other conditions which are often the first choice of diagnosis during initial examinations. Confirmation can also be a challenge, so differential diagnosis of prolactinomas in particular must be considered, as follows: (i) Esthesioneuroblastoma (ENB), which are rare, malignant neoplasia that originate in the olfactory neuroepithelium. They typically grow toward the upper nasal cavity and may extend into the paranasal sinuses or intracranial region. ENBs are characterized by their neuroectodermal origin and can display a wide range of histological patterns [13]; (ii) Meningiomas, which are typically benign neoplasms arising from the meninges. They are derived from meningothelial cells and can occur at many locations. Meningiomas are often slow-growing tumors that compress adjacent structures, leading to neurological symptoms [14]; (iii) Chordomas, which are rare, locally aggressive tumors derived from remnants of the embryonic notochord. They typically occur in the axial skeleton, particularly the skull base, spine, and sacrum. Chordomas exhibit histological features resembling notochordal tissue and are often slow-growing but locally invasive [15]; (iv) Nasopharyngeal carcinoma (NPC), which is a malignancy arising from the epithelial cells lining the nasopharynx. It is strongly associated with Epstein-Barr virus (EBV) infection and exhibits distinctive histological subtypes, including keratinizing squamous cell carcinoma, non-keratinizing carcinoma, and undifferentiated carcinoma [16]; and (v) Cranial nerve schwannomas, which are benign neoplasms originating from Schwann cells surrounding cranial nerves. They most commonly affect the vestibular portion of the eighth cranial nerve (vestibulocochlear nerve) [17,18].

In 2022, the 2021 World Health Organization Classification of Central Nervous System Tumors [19] and 2022 World Health Organization Classification of Endocrine and Neuroendocrine Tumors [20] made changes in the categorization of pituitary adenomas as neuroendocrine tumors and proposed the name to be revised to pituitary neuroendocrine tumor (PitNET). The previous classification for this tumor was “0” for benign tumors [17-19]. In contrast, the fifth edition of the WHO classification changed this code to “3” for primary malignant tumors, the same as neuroendocrine tumors in other organs [18]. This update made changes in the pathology and radiology field regarding pituitary adenomas; however, in clinical and treatment practices, no additional assistance or direction was given [19,20].

## Conclusions

This case exemplifies a patient who initially presented with a prolactin level lower than 20 Ng/ml and, after surgical resection, began to exhibit values above 450 Ng/ml. Given the difficulty encountered in the initial evaluation, it is crucial that in similar cases, endocrinological evidence is also sought.

The hook effect is a significant phenomenon to consider in the diagnosis and monitoring of giant and invasive prolactinomas. Understanding this effect and adopting alternative testing methods are essential to avoiding misdiagnoses and ensuring proper evaluation of hormonal status in these cases.
